# Impact of Biochar and Bioorganic Fertilizer on Rhizosphere Bacteria in Saline–Alkali Soil

**DOI:** 10.3390/microorganisms10122310

**Published:** 2022-11-22

**Authors:** Yin-Yu Gu, Hai-Yang Zhang, Xiao-Yan Liang, Rao Fu, Meng Li, Chuan-Jie Chen

**Affiliations:** Shandong Institute of Sericulture, Shandong Academy of Agricultural Sciences, Yantai 264002, China

**Keywords:** biochar, bioorganic fertilizer, rhizosphere bacteria, saline–alkali soil

## Abstract

Biochar and bioorganic fertilizers (BOF) that are used in agriculture can, both directly and indirectly, impact rhizosphere soil microorganisms. However, changes to the halophyte rhizosphere bacterial community after applying biochar and BOF to saline–alkali soil have not been thoroughly described. This study has investigated the bacterial communities of halophytes in saline–alkali soil through the addition of different biochar and BOF formulas using Illumina-based sequencing of the 16S rRNA gene fragment. B_BOF (biochar and BOF combined application) had the best effect, either by promoting the plant growth or by improving the physical and chemical properties of the soil. The concentration of the rhizosphere bacterial communities correlated with the changes in soil organic matter (OM) and organic carbon (OC). Proteobacteria, Actinobacteria, Chloroflexi, and Acidobacteria accounted for >80% of the total bacteria in each treatment. In addition, the abundance of *Micromonospora* was much higher in response to B_BOF than to the other treatments. BOF, with or without biochar, significantly influenced the bacterial community composition in the saline–alkali soil. The OC, OM, total nitrogen, and the available phosphorus had significant effects on the bacterial structure of this soil. The complex correlation of the bacterial communities between CK and B_BOF was higher compared to that between CK and FB or between CK and BOF. These findings suggested that the plant growth, the soil characteristics, and the diversity or community composition of the rhizosphere bacteria in saline–alkali soil were significantly influenced by B_BOF, followed by BOF, and then biochar; fine biochar had a stronger effect than medium or coarse biochar. This study provides an insight into the complex microbial compositions that emerge in response to biochar and BOF.

## 1. Introduction

Soil salinity is one of the most widespread threats to the sustainable development of agriculture [1]. In addition to naturally caused saline land, much arable land gradually becomes salinized due to either improper water or land management practices [2,3]. Seven percent of the world’s arable land is impacted by salinity, which greatly threatens the global food supply [4], including about 7.6 million hm^2^ of cultivated saline–alkali land in China [5]. The Yellow River Delta, which is one of the three bayou deltas in China, is characterized by high soil salinity and poor soil fertility due to the interaction between sea and land and has great potential for land exploration and use [6]. An increasing number of articles on the reclamation of saline–alkali land have mainly dealt with water conservancy engineering measures, such as salt drainage by hidden pipes, physical measures, such as sand mixing and mulching, chemical improvement measures, such as adding cow manure, gypsum, and straw, and biological improvement measures, such as planting saline–alkali tolerant plants [7,8,9]. Among them, biochar and bioorganic fertilizers (BOFs) have been proven as effective measures to improve saline–alkaline soil quality [10,11].

As a carbon-rich material, biochar is produced from biomass raw materials, such as crop residues, animal wastes, or wood, under anoxic conditions during a process called pyrolysis [12] and is a versatile source of renewable energy with the potential to generate heat, electricity, and liquid biofuels [13]. With the improvement of the physical, chemical, and biological properties of soil, biochar shows great potential as an effective and ecological amendment to the soil, especially degraded coastal saline soil [14]. As a porous material with a strong absorption ability, a huge surface area, and a low bulk density [3], biochar could bring multiple benefits when it is applied to soils, including enhanced carbon sequestration [15], improved soil fertility and quality [16], reduced greenhouse gas emission [13], changes to the soil microbial structure [17], and increased plant growth [18]. In the meantime, studies on biochar in saline land reclamation are also attracting increasing attention. They indicate improved saline soil physicochemical properties [19] and increased biomass [20]. In conclusion, research on the application of biochar in agriculture has become more and more extensive, and the positive effects in degraded soils are becoming better known.

Bioorganic fertilizers (BOFs) are fertilizers that are composed of microorganisms with specific functions and organic materials (such as the solid-state fermentation of agro-industrial waste) [21]. Previous studies have shown that BOF is a safe and effective alternative for chemical fertilizer [22]. It can improve the supply of plant nutrients [23], increase the yield [24], and enhance crop disease and stress resistance [25]. BOFs also aid in the secretion of plant growth hormones and help to counteract the negative effects of chemical fertilizer [26]. Thus, the use of BOFs is an important measure to protect the ecological environment and to promote the inevitable trend of the sustainable development of agriculture. [27]. In addition, some researchers have reported that BOFs are effective in improving saline soils [28], such as by promoting the soil microecological environment of saline–alkali land [29] and enhancing nutrient buffering in order to prevent salt build-up [11]; however, much less attention has been given to the combined use of BOFs and biochar for the reclamation of saline–alkali land.

Salt-tolerant plants are preferred for saline reclamation because they can achieve normal growth. *Mesembryanthemum crystallinum* Linn. is a medicinal and edible halophyte that originates from South Africa [30,31], while *Aptenia cordifolia* (L. f.) Schwant, which is also known as *Mesembryanthemum cordifolium* L. f., is a salt-tolerant succulent with potential medicinal value that is endemic to South Africa [32,33,34]. *M. crystallinum* and *A. cordifolia* were used in this study in order to examine the effect of biochar and BOF on plant growth, soil physicochemical properties, and soil bacteria communities in saline–alkali land and to explore the feasibility of using these components to improve the soil.

## 2. Materials and Methods

### 2.1. Biochar and BOF Preparation

The BOF was purchased from Yangfeng Agricultural Technology Co., Ltd. (Weifang, China), which uses humic acid, mushroom residue, corn residue, and soybean meal as the main substrates and supplements these with *Bacillus subtilis*, *B. licheniformis*, and *B. mucilaginous* (among them, the effective bacteria is ≥500 million/g), OM ≥ 60%, nitrogen, phosphorus, and potassium content ≥ 6–8%. The biochar was purchased from Taiyu Bioengineering Co., Ltd. (Qixia, China). The biochar sample was made from apple stems and was charred at 450 °C for 24 h. The sample was then milled to pass through 10, 30, and 60 mesh sieves, after which the pH was 7.49, 7.36, and 7.45, respectively, and the electrical conductivity (EC) was 0.357 mS/cm, 0.355 mS/cm, and 0.349 mS/cm, respectively. The larger meshed samples contained the smaller mesh samples.

### 2.2. Field Experiments

The field experiments were carried out at the Institute of Modern Agriculture on the Yellow River Delta, Shandong Academy of Agricultural Sciences (118.37° N, 37.17° E), which is located in Dongying, China. The experimental area was a saline–alkali wasteland, which was used for this experiment after planting spinach for one year with no fertilizer. The soil was classified as silty clay [10]. All biochar and fertilizer application rates used in the experiments were 150 t/ha and are hereafter referred to as CK (without biochar and BOF), CB (10 mesh biochar), MB (30 mesh biochar), FB (60 mesh biochar), BOF (bioorganic fertilizer), and B_BOF (60 mesh biochar + bioorganic fertilizer). The biochar and BOF were spread on the field in November and the soil samples from each plot were taken in March before planting. *M. cordifolium* and *A. cordifolia* seedlings were transplanted into the field at the four-leaf stage in April and no field management was performed, except watering. The plant biomasses were quantified in June. Three plants were randomly selected from each treatment and the aerial parts of each plant were harvested, along with the rhizosphere soil samples. The fresh weight (FW) of the aerial part of each plant was recorded. Three plants were selected randomly from each treatment, the over-ground part was harvested separately, and rhizosphere soil was collected at the same time. The excess soil was removed from the root by shaking, and only tightly adherent soil remained for study. The rhizosphere soils of the three plants in each treatment were removed from the topsoil (0–20 cm) and mixed evenly, the impurities were removed, and the samples were divided into two parts. A portion was immediately snap-frozen in liquid nitrogen and sent on dry ice to MajorBio for high-throughput sequencing, while the remainder was air-dried, homogenized, and again sieved (<2 mm) to remove any residue of silica sand or plant roots in order to determine the soil’s physicochemical properties. All experiments were conducted in triplicate.

### 2.3. Soil Physicochemical Analysis

The soil pH values were determined using a Shanghai Lei Magnetic Multi-Parameter Water Quality Analyzer DZS-708. The EC was measured at a soil depth of 10 cm using a FieldScout EC450 meter. The total phosphate (TP) content was measured using ICP-MS after microwave digestion (MARS5, CEM, Matthews, NC, USA) (0.1 g sample + 6 mL concentrated nitric acid). Available phosphorous (AP) was extracted with 0.5 M NaHCO_3_ and was measured using a segmented continuous flow analyzer (Quaatro, Bran+Luebbe, Norderstedt, Germany). Alkaline nitrogen (AN) and TN contents were determined using an elemental analyzer (FLASH-2000, Thermo Scientific, Waltham, MA, USA). The OM was quantitated using the potassium dichromate oxidation method and OC was determined using an SSM-5000A (Shimadzu, Kyoto, Japan) carbon analyzer [10].

### 2.4. Soil Microbial Community Analysis

The genomic DNA from the microbial community was extracted from the rhizosphere soil samples using the E.Z.N.A.^®^ soil DNA Kit (Omega Bio-Tek, Norcross, GA, USA) according to the standard protocol, and a NanoDrop 2000 UV-vis spectrophotometer was used to check the quality of the extracted DNA. The bacterial universal V3-V4 region of the 16S rRNA gene was amplified using the primers 338F (50-ACTCCTACGGGAGGCAGCAG-30) and 806R (50-GGACTACHVGGGTATCTAAT-30). The PCR mixtures contained 4 μL of 5× *TransStart*FastPfu buffer, 2 μL of 2.5 mM dNTPs, 0.8 μL of each primer (5 μM each), 0.4 μL of *TransStart*FastPfu DNA Polymerase, and 10 ng template DNA, adding ddH_2_O to a final volume of 20 μL. All reactions were performed in triplicate. The PCR cycling conditions included an initial denaturation at 95 °C for 3 min, 27 cycles of denaturing at 95 °C for 30 s, annealing at 55 °C for 30 s, and extension at 72 °C for 45 s, followed by a single extension at 72 °C for 10 min and a continued hold at 4 °C. The resulting PCR products were extracted from 2% agarose gels and quantified using a Quantus™ Fluorometer (Promega Corporation, Madison, WI, USA) after purification. The purified amplicons were pooled in equimolar ratios and paired-end sequenced by Majorbio Bio-Pharm Technology Co., Ltd. (Shanghai, China) using an Illumina MiSeq PE300 platform (Illumina Inc., San Diego, CA, USA). Raw 16S rRNA gene sequencing reads were demultiplexed, quality-filtered with fastp version 0.20.0 [35], and merged by FLASH version 1.2.7 [36]. The operational taxonomic units (OTUs) with a 97% similarity cut-off were clustered using UPARSE v.7.1, and chimeric sequences were identified and removed. The taxonomy of each OTU representative sequence was analyzed with RDP Classifier v.2.2 against the 16S rRNA database using a confidence threshold of 0.7. The complete sequences generated in this study are available in the NCBI SRA database under accession number SRA data: PRJNA863446.

### 2.5. Data Analysis

The calculations were performed using Microsoft Excel, and the statistical analyses of all parameters were performed using DPS Statistics 18.10 software (http://www.dpsw.cn, accessed on 6 August 2022). All analyses, including α-diversity, β-diversity, and network structure, were performed on the Majorbio Cloud Platform (www.majorbio.com, accessed on 16 August 2022) and α-diversity was calculated using Mothur software (v.1.30.2). Rarefaction curves were generated based on the observed species richness using Mothur at a 97% identity level. Venn and bar diagrams were generated using R script (v.3.3.1), and Circos was visualized using Circos-0.67-7 (http://circos.ca/, accessed on 16 August 2022). Beta diversities were visualized using principal coordinates analysis (PCoA), based on the distance matrix, with bray_curtis. RDA was analyzed using R (version 3.3.1) RDA and graphed using the vegan package, and VPA (variance partitioning analysis) was analyzed by an analysis of VPA in R language vegan package. Finally, a network analysis was performed to explore the complexity of the interactions among the microbial taxa using Networkx software. Data are presented as the means and standard errors. Differences between the means of different treatments were determined using the Duncan test at *p* < 0.05.

## 3. Results

### 3.1. Biomass of M. crystallinum and A. cordifolia

**Figure 1:**
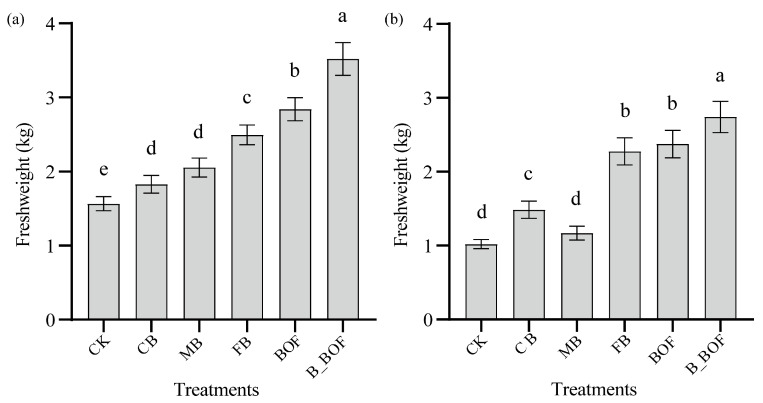
Effect of biochar and BOF on *M. crystallinum* and *A. cordifolia* fresh biomass. CK: soil without amendment; CB: soil amended with 10 mesh biochar; MB: soil amended with 30 mesh biochar; FB: soil amended with 60 mesh biochar; BOF: soil amended with BOF; B_BOF: soil with 60 mesh biochar and BOF. Error bars represent standard errors of the means (n = 3). Different lowercase letters indicate significant difference among treatments (*p* < 0.05). (**a**) *M. crystallinum*, (**b**) *A. cordifolia*.

Significant increases in the FW were observed in all of the treatment plots, except for the MB of *A. cordifolia* (Figure 1 and Figure S1), regardless of whether the seedlings were *M. crystallinum* or *A. cordifolia*, and whether biochar and BOF were applied alone or in combination. For both *M. crystallinum* and *A. cordifolia*, the FW was highest following the B_BOF application, followed by the BOF and FB. The results from the current study showed an increase of 25–79% following the biochar application alone and an increase of 92.73–106.76% under the BOF application alone, compared to CK; furthermore, there was a 13.71–44.67% increase after B_BOF amendment, compared to biochar alone, and an increase of 5.67–15.35% compared to BOF alone (Table S1).

### 3.2. Soil Physicochemical Properties

The effect of biochar and BOF on the physical and chemical properties of soil is shown in Table 1. There was no significant difference in the soil pH caused by the treatment type. All of the treatments, except for CB, significantly reduced the EC versus the CK. The TP and the AP were significantly higher following the B_BOF, BOF, and FB treatments than the MB, CB, and CK treatments. The B_BOF and the FB significantly promoted the TN content, compared to CK, with the highest levels produced by B_BOF. The B_BOF also induced the highest AN content of all of the treatments. The OC and the OM of all of the plots receiving biochar (B_BOF, FB, MB, and CB) were significantly higher than those without (CK and BOF), and B_BOF promoted the highest OC and OM levels.

### 3.3. Sequence Data and α-Diversity Index Analysis

After processing, 891,819 high-quality sequences remained, with an average length of 415 bp across all of the 18 samples, and were investigated after read-quality filtering. The total number of bases was 370,190,141. The rarefaction curves tended to approach the saturation plateau in all of the 18 samples (Figure S2), combined with the estimated coverage values (Table S2), demonstrating that the data were sufficiently large enough to capture most of the bacterial diversity in the samples. The number of OTUs obtained was the highest in the MB treatment and the lowest in the B_BOF treatment. The indices of sobs, ace, Shannon, etc., showed that the diversity and the richness of the B_BOF were lower compared with the other treatments.

**Figure 2:**
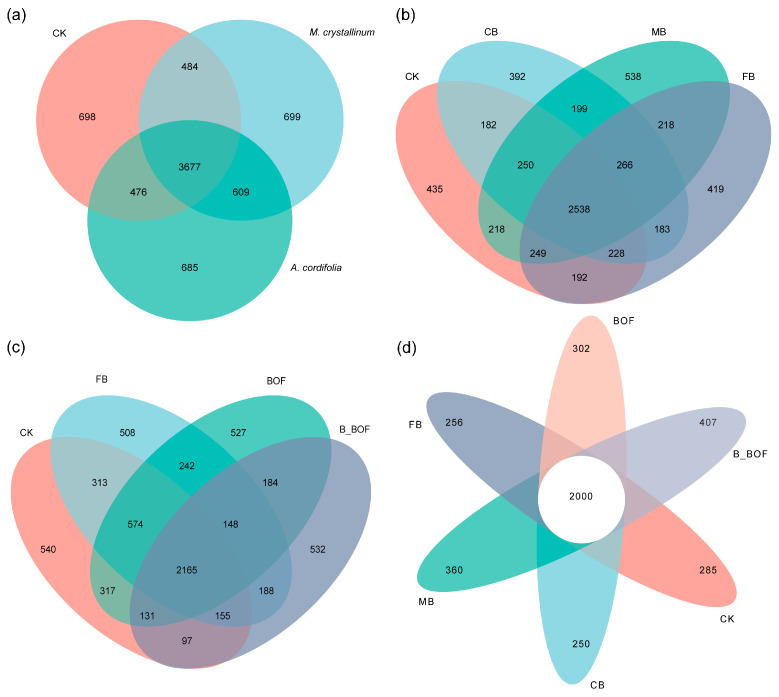
Venn diagrams of the number of OTUs obtained in different treatments. (**a**) Grouping by plants. (**b**) Grouping by different biochar. (**c**) Grouping by combined application of FB and BOF versus independent application. (**d**) Grouping by different treatments of biochar and BOF.

The number of common and unique bacterial OTUs in the different samples is shown in Venn diagrams (Figure 2). A total of 7328 OTUs were detected across all of the libraries, with 3677 OTUs common to the different plant samples (Figure 2a). The different particle sizes of the plots receiving the biochar shared 2538 OTUs of the total 6507 OTUs, with the highest unique OTUs (n = 538) in the MB, and the lowest unique OTUs (n = 392) in the CB (Figure 2b). In addition, CK, FB, BOF, and B_BOF harbored 540, 508, 527, and 532 unique OTUs, respectively, and they shared 2165 OTUs (Figure 2c). Additionally, all of the treatments shared 2000 OTUs, with a higher number of unique OTUs being obtained in the B_BOF samples than in the CK, CB, MB, FB, and BOF samples (285, 250, 360, 256, and 302, respectively) (Figure 2d).

### 3.4. Microbial Taxonomic Analysis and Core Genus Distribution

**Figure 3:**
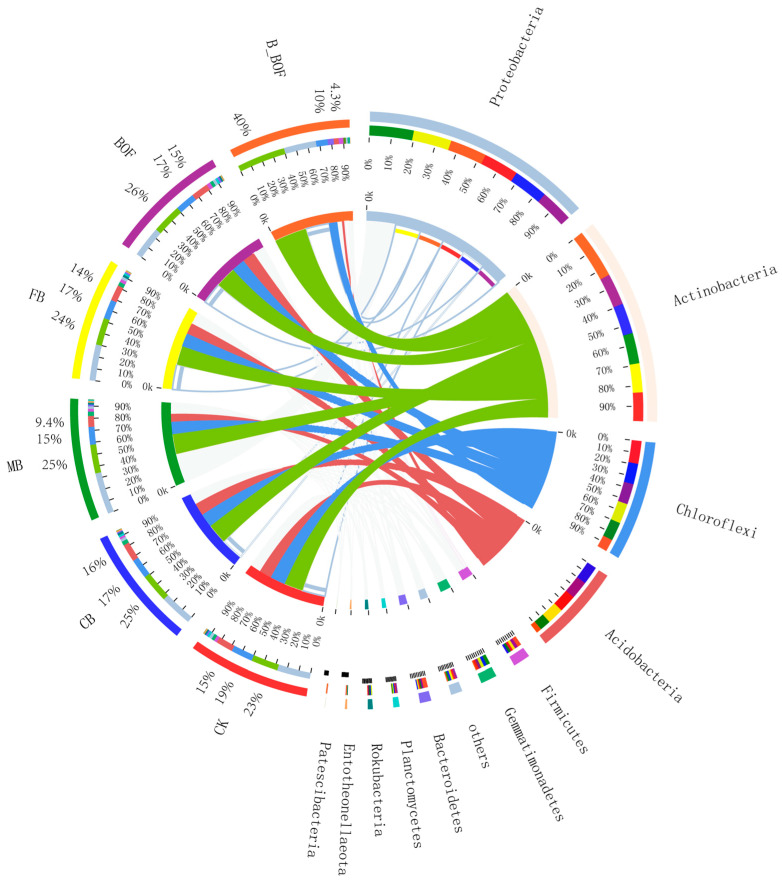
Relative abundance of bacteria in each sample at the phylum level. The green band is Actinobacteria, the red band is Acidobacteria, and the blue-purple band is Chloroflexi.

The 7328 OTUs were classified into 37 phyla, 101 classes, 271 orders, 501 families, 1054 genera, and 2221 species. The bacterial composition and the relative abundances varied across the different samples. High-throughput sequencing revealed the diversity of the bacterial communities in the different samples at the phylum level (Figure 3). The dominant bacterial phyla were Proteobacteria and Actinobacteria, accounting for more than 50% across all of the samples, followed by Chloroflexi. The dominant phylum in the B_BOF samples was Actinomycetes, while the dominant phylum in the other treatment groups was Proteobacteria. The B_BOF harbored 40.38% Actinobacteria, which was significantly higher than the other samples, and 10.2% Choloroflexi and 4.3% Acidobacteria, which was significantly lower than the other samples.

**Figure 4:**
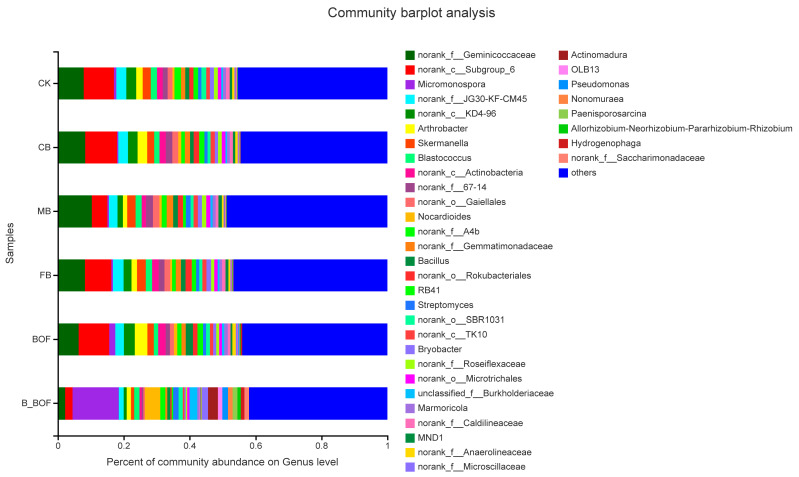
Relative abundance of the predominant genera (top 40) in each sample. Taxa with an abundance < 0.01 are included in “others”. The *x*-axis represents the relative abundance of all communities, and the *y*-axis represents different communities.

The clustering of the top 38 genera is shown in Figure 4 and Table S3. The distribution of the genera differed significantly across the different samples and belonged to five phyla, including Actinobacteria (12 genera), Chloroflexi (9), Proteobacteria (7), Acidobacteria (3), Firmicutes (2), Gemmatimonadetes (1), Bacteroidetes (1), Rokubacteria (1), and Patescibacteria (1). *Norank_f_Geminicoccaceae* was the predominant genus in the FB and the MB samples, *norank_c_Subgroup_6* was the predominant genus in the CK, the CB, and the BOF samples, and *Micromonospora* was the most abundant genus in the B_BOF samples. The co-application of biochar and BOF increased the abundances of *Hydrogenophaga* and *norank_f_Saccharimonadaceae* by different degrees, regardless of the particle size, or the presence or absence of added fertilizers. However, the abundance of *norank_f_A4b*, *norank_o_SBR1031*, and *norank_f_Caldilineaceae* decreased, compared to the CK, regardless of the particle size, or the presence or absence of added fertilizers.

**Figure 5:**
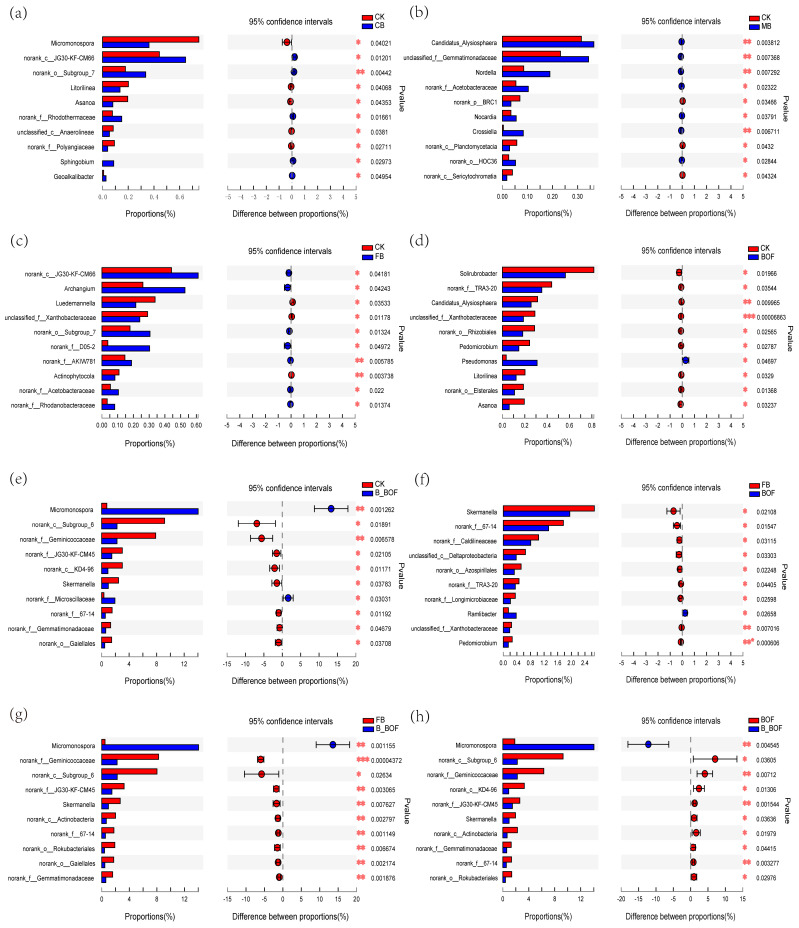
Comparison of the abundance of the top 10 dominant bacterial genera in the different samples. *** indicates a significant difference of *p* < 0.001, ** *p* < 0.01, and * *p* < 0.05. The *x*-axis represents the mean proportion of the genus, and the *y*-axis shows the top 10 dominant bacterial genera. (**a**) Grouping by CK and CB. (**b**) Grouping by CK and MB. (**c**) Grouping by CK and FB. (**d**) Grouping by CK and BOF. (**e**) Grouping by CK and B_BOF. (**f**) Grouping by FB and BOF. (**g**) Grouping by FB and B_BOF. (**h**) Grouping by BOF and B_BOF.

The relative abundance of the core genera was also compared, and the top 10 are shown in Figure 5. The relative frequencies of *Micromonospora* and *norank_f_Microscillaceae* were higher in the B_BOF than in the CK samples, while the frequency of *norank_o_Gaiellales* was lower in the B_BOF than in the CK samples. The relative abundance of *Pseudomonas* (*p* = 0.047) was significantly higher in the BOF than in the CK samples. Seven genera were higher in the FB than in the CK samples, *norank_c_JG30-KF-CM66*, Archangium, *norank_o_Subgroup_7*, *norank_f_D05-2*, *norank_f_AKIW781*, *norank_f_Acetobacteraceae*, and *norank_f_Rhodanobacteraceae*. In the MB sample, *Cdidatus_Alysiosphaera*, *unclassified_f_Gemmatimonadaceae*, *Nordella*, *norank_f_Acetobacteraceae*, *Nocardia*, *Crossiella*, and *norank_o_HOC36* were higher than that in the CK samples, while *norank_c_JG30-KF-CM66*, *norank_o_Subgroup_7*, *norank_f_Rhodanobacteraceae*, *Sphingobium*, and *Geoalkalibacter* were higher in the CB than in the CK samples. *Micromonospora* was much higher in the B_BOF samples than in the BOF and FB samples, while *Ramlibacter* was higher in the BOF than in the FB samples.

### 3.5. β-Diversity Analysis

**Figure 6:**
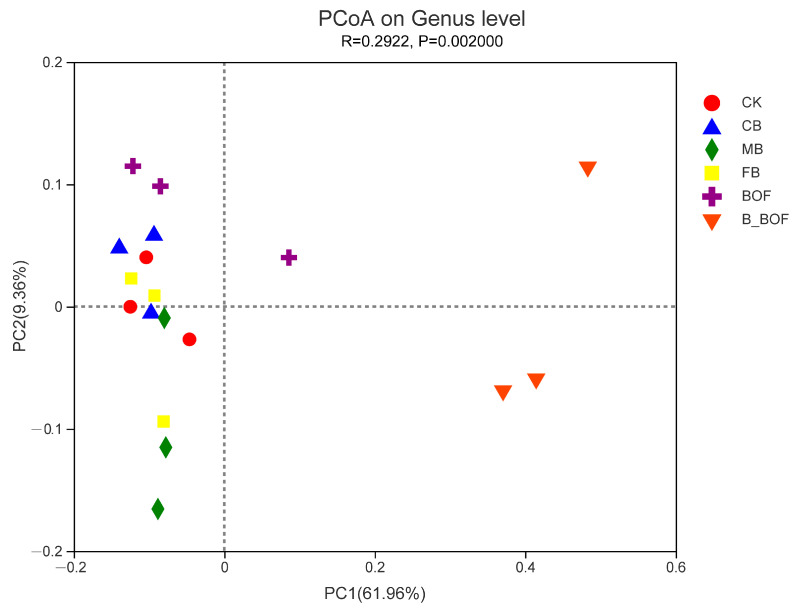
PCoA plot of the relationship between the samples based on similarity in the community composition. Two first components (PCoA 1 and PCoA 2) were plotted and represent 71.32% of the variation.

In order to further identify the microbial population that was associated with biochar and BOF, PCoA was conducted in order to determine the extent of the rhizosphere soil population variation by treatment type (Figure 6). The clustering of the samples that were treated with biochar of different particle sizes and CK indicated that the community structures were similar among them. In contrast, there were significant differences in the community structures of the samples that were treated with the BOF, the B_BOF, or the CK, especially between the B_BOF and the other treatments. These findings indicated that the BOF and B_BOF treatments were associated with unique bacterial community structures. PCoA identified two principal component factors that were related to the percentage of abundance of the groups, explaining 61.96% (PC1) and 9.39% (PC2) of the variation, respectively. The highest variation in the microbiota associated with the different samples (61.96%) represented a strong separation based on the B_BOF, while the BOF was associated with a higher PCoA 2 value (9.39%). The PCA analysis revealed that the BOF treatment shifted the bacterial communities, and the B_BOF was a strong factor that was associated with variation in the community composition.

### 3.6. Relationship between Environmental Parameters and Microbial Communities

**Figure 7:**
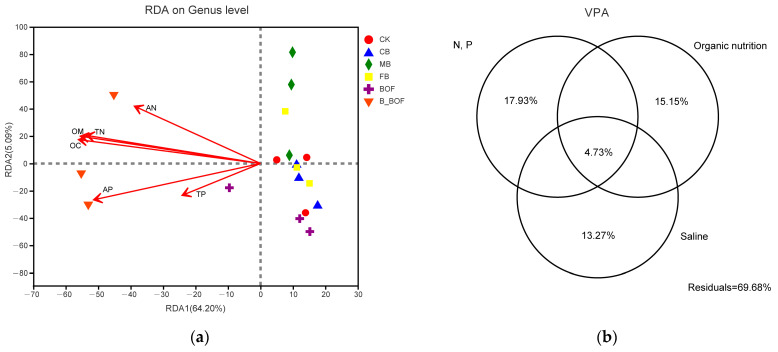
Relationship between soil characteristics and soil bacterial community. (**a**) RDA, two first components (PCoA 1 and PCoA 2), which represented 69.29% of the variation, were plotted. (**b**) VPA, variation partitioning (% variation explained) of rhizosphere bacterial community in relation to combined environmental variables.

RDA was conducted in order to examine the relationship between the environmental parameters and the composition of the microbial communities (Figure 7a). For the microbial community composition, the first axis accounted for 64.20% of the overall variation, while the second axis accounted for 5.09%. The RDA showed that the bacterial community structure that was associated with the B_BOF treatment was positively correlated with the AP, the TN, the OC, and the OM. The VPA showed that the saline (pH and EC), the organic nutrition (OC and OM), and the nutrition (N and P) factors explained 13.27%, 15.15%, and 17.93% of the total variance in the rhizosphere bacterial communities, respectively (Figure 7b). Meanwhile, a joint environmental effect (4.73%) explained a lower proportion of the variation in the rhizosphere bacterial turnover. Among these variables, N and P had a large influence on governing the bacterial turnover in the rhizosphere. 

### 3.7. Network Structure

**Figure 8:**
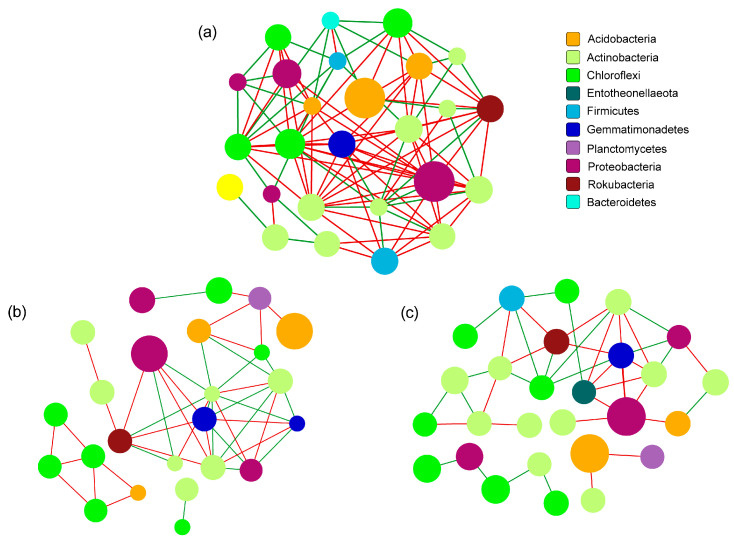
Correlation network analysis of microbial communities. Node color represents phylum classification. The size of the node is proportional to the richness of bacteria. Edge color corresponds to positive (red) and negative (green) correlation, and the edge thickness is equivalent to the correlation values. (**a**) The network between CK and B_BOF. (**b**) The network between CK and BOF. (**c**) The network between CK and FB.

Correlation network analysis was conducted in order to explore the complexity of the interactions within the communities following the different treatment types and to assess their topological properties. The findings showed that there was a difference between the communities based on the addition of biochar and BOF (Figure 8). The complexity and the modular structures were higher following the CK–B_BOF treatment (between CK and B_BOF) than the CK–FB (between CK and FB) or the CK–BOF (between CK and BOF) treatment. Specifically, the average number of connections per node was higher following the CK–B_BOF treatment (node average degree = 8) than the CK–FB (node average degree = 3.11) or the CK–BOF (node average degree = 3.75) treatment (Table S4). The CK–B_BOF treatment also resulted in a higher number of positive correlations (positive edges = 67) than the CK–FB (positive edges = 23) or the CK–BOF (positive edges = 26) treatment. The positive edges were higher following CK–FB treatment than CK–BOF treatment, albeit with the same number of negative correlations. The nodes with the highest connections were norank_f_JG30-KF-CM45 in CK–B_BOF, with a degree of 14, *Micromonospora* in CK–BOF, with a degree of 10, and *Norank_f_Gemmatimonadaceae* and *norank_f_67-14* in CK–FB, with a degree of 7 (Table S5).

## 4. Discussion

The response of plants to biochar amendments in the soil varies enormously based on differences in the soil type, the biochar properties, and the biochar application rates, methods, and frequencies [37]. Additionally, biochar is often used in combination with inorganic fertilizers, organic fertilizers, and PGPRs in agricultural research and significantly promotes plant growth [38,39]. Similarly, the above-ground biomass in the current study was enhanced, regardless of the biochar or BOF application alone, and the results of the BOF were better than those of the biochar; furthermore, the combined application of the two was more effective than the individual application. This is similar to the findings by Saxena et al., who reported that the addition of biochar to soil induced the overall growth of plants, but inoculation with *Bacillus* sp. enhanced the growth even further [38]. One reason for the increased effectiveness of B_BOF may be that the direct source of nutrients that the B_BOF brings to the soil was more than that of biochar or BOF alone. Another reason may be the reduced nutrient leaching of B_BOF, owing to the fact that more nutrients can be adsorbed to biochar’s surface and be retained in the soil; additionally, the refuge for microorganisms that was provided by the porosity of biochar, combined with the abundant nutrients and bacteria of the BOF, aided the survival of microbial species, which was beneficial to the growth of the plants. In the current study, no significant difference was observed among the treatments, regardless of the biochar particle size and whether BOF was added, which was partially identical to You et al. [37]. This may be due to the pH of the biochar that was used in the current study being lower than that of the soil, or the secretion of organic acids from the stimulated roots of the plants receiving the fertilizer treatment [40]. The EC of soil was shown to vary significantly in response to biochar application in different studies [41,42,43]. The EC may have decreased in this study because salt-tolerant plants grow better in response to biochar and BOF, or because different raw materials or preparation methods were used in the biochar manufacturing process. Additionally, there were no significant differences in the EC for all of the treatments, except for the control, which indicated that the effect of biochar and BOF application alone, or combined, in reducing the soil EC is similar. The effects of biochar on plant-available P in soils vary [44], but most studies suggest that biochar can increase the availability and the uptake of P in soils by acting as a direct source of P and indirectly improving the conditions of the medium [2]. In the current study, P was significantly higher in response to the BOF than the biochar alone or the CK, and there were no differences in the CK, the MB, or the CB. The B_BOF induced the highest AP content on the basis of the same TP content, which may be an important factor in promoting plant growth. The biochar effects on N are also inconsistent [45,46]. The co-application of biochar and BOF significantly increased both the TN and the AN content, which may be related to biochar feedstock and the ability of organic fertilizer and biochar to increase N compared to biochar alone [45]. Biochar amendment significantly increased the content of the OC and OM, which is consistent with the results of previous studies [41,46,47,48,49]. However, the addition of BOF had no effect, while B_BOF led to the greatest improvement of all of the treatments. N and P explained a higher proportion of the variation in the rhizosphere bacteria compared to the saline and organic nutrients, which may be owing to the fact that N and P are the main nutrients and limiting factors for plant growth [50], which in turn affected the bacterial changes.

Recent studies indicate that the addition of biochar, BOF, or both in combination, effectively impacts the soil microorganisms, which play a critical role in nutrient recycling, the suppression of pests and diseases, and the promotion of plant growth [13,21]. The richness of B_BOF was significantly lower than that of the other treatments, suggesting that the co-application of biochar and BOF greatly altered the composition of the bacterial communities, which may be due to the blooms of the dominant species affecting the growth of the rare species. Although there were no differences in the unique OTUs among the plant treatments and the CK, the number of OTUs shared by both of the plants was higher than that shared between each plant and the CK. This indicated that *M. crystallinum* and *A. cordifolia* both affected the soil microbial composition in similar ways. Dominant phyla vary with soil properties and additives [51,52]. It can be seen that, in our study, Proteobacteria, Actinobacteria, Chloroflexi, and Acidobacteria, were the top four most abundant phyla, which is partly consistent with prior research. You et al., reported that Bacteroidetes and Proteobacteria were the predominant phyla in the rhizosphere soil of *M. crystallinum* [37]; however, according to Huang et al., Proteobacteria, Chloroflexi, and Acidobacteria were the three most abundant phyla following the application of biochar under salt-stress treatment [5]. Actinobacteria are often associated with the degradation of recalcitrant polymers and are thus considered to be ecologically important for the turnover of the OM in soil [53], and the relative abundance of Actinobacteria increases with biochar amendment [54]. Compared to the CK group, the relative abundance of Actinobacteria was slightly higher following the treatment with biochar, but the difference was not significant. However, the relative abundance of Actinobacteria was significantly higher following the treatment with B_BOF than that following the CK treatment, which may be related to the higher soil nutrients of B_BOF, as RDA also showed that the relative bacterial abundance after the B_BOF application correlated positively with the TN, the AP, the OC, and the OM, which is partly consistent with previous studies [11]. Chloroflexi is an oligotrophic bacterium that survives in low-nutrient soil, while Proteobacteria prefers nutrient-rich soil [5]. This may explain the low abundance of Chloroflexi in the B_BOF-treated soil, and the high abundance of Proteobacteria in the MB-, BOF-, and B_BOF-treated soil. Interestingly, the bacterial community of CK–B_BOF is more complex than that of CK–FB or CK–BOF, in other words, compared to the FB or BOF group, the application of the B_BOF enhanced and elevated the complexity of the microbial networks and reshaped the network core microorganisms and hubs, which deserves further study. 

*Pseudomonas* is a well-known bacterium that is used as plant-growth-promoting rhizobacteria (PGPR) because of its ability to protect plants from disease and increase the availability of phosphates [55,56]. The fertilizer effect can be augmented by the application of *Pseudomonas* and biochar in order to alleviate salt stress [57]. This may explain the much higher relative abundance of *Pseudomonas* in BOF than in CK. Being a denitrifier and pesticide degrading bacterium [58,59], the abundance of *Hydrogenophaga* was higher following the application of biochar and BOF in different degrees, regardless of the particle size or the presence of additional fertilizers, which is consistent with the findings of Guo et al. and You et al. [58,59]. The same increase was seen for *norank_f_Saccharimonadaceae*, which can degrade hydrocarbons, especially following B_BOF treatment [60]. These could benefit the soil nutrients and the plant growth. *Bacillus* is well known as an effective biological control bacterium [38]. Wang et al. demonstrated that biochar soil amendment enriched this bacterium [61]. It is worth noting that the presence of *Bacillus* in soil does not necessarily increase with the addition of *Bacillus*. Indeed, Zhu et al., found that the *Bacillus* concentration in soil did not increase after the application of *Bacillus* compound biofertilizer [62]. In the current study, the relative abundance of *Bacillus* in the BOF-treated soil was >80% higher than that in the CK-treated soil. *Bacillus* was also higher following FB and MB treatment than CK treatment but was lower following B_BOF treatment than CK treatment. BOF contains *Bacillus*, therefore it is not surprising that the amount of *Bacillus* increased in the BOF-treated soil. The FB and MB findings are consistent with those that were reported by Wang et al. [61]. The inability of B_BOF to induce the growth of *Bacillus* may be explained by the competitive relationship between *Bacillus* and other bacteria in the treated soil. While the effect of BOF on *Bacillus* growth was unexpected, the effect on the *Micromonospora* growth was way beyond expectations. *Micromonospora* was the most abundant genus in the B_BOF-treated soil and was much higher in both the B_BOF-treated and BOF-treated soil than in the CK-treated soil. This result is consistent with the findings of Deng et al., who reported enhanced *Micromonospora* growth under the biochar and microcapsule treatment of phenanthrene polluted soil [63]. According to Li et al., *Micromonospora* has plant-growth-promoting traits, including nitrogen fixation and the inhibition of plant pathogens, therefore, it may aid B_BOF- and BOF-induced plant growth [64]. *Gaiella* and *norank_o_Gaiellales* are both Actinobacteria, which promote plant growth by increasing the nutrient availability and assimilating and enriching the beneficial bacteria. *Norank_o_Gaiellales* contributes to the ability of ramie to tolerate poor soil [65]. Interestingly, the relative abundances of *Gaiella* and *norank_o_Gaiellales* alone were higher in the biochar-only treatments than in the CK treatments, regardless of the biochar particle size. However, when BOF was present, the abundance was lower than CK. *Norank_f_Geminicoccaceae* was the predominant genus in the FB and the MB; however, the reason for this finding requires further study. Overall, the biochar and the BOF were able to directly recruit beneficial bacteria through their intrinsic ability to improve the soil conditions for plant growth.

The particle size of the biochar is another important factor affecting the soil characteristics and functions that has received minimal research attention [66,67]. With the exception of AN, the FB performed best when the soil N and P indicators were involved, and the biochar amendment significantly increased the content of the soil OC and OM, regardless of the particle size, although there were no significant differences between the treatments that used different biochar particle sizes. The different plants responded slightly differently to the biochar particle size, but the trend was similar, with fine biochar inducing the best response. The results of the current study have confirmed the conclusion of Liu et al. and Gu et al. that fine biochar is superior to coarse biochar [49,68]. The larger internal surfaces and the porous structure of the finer particle size biochar should be responsible for this conclusion, as these factors result in lower soil volumetric weight and higher soil aeration [49] and cause the biochar to be more effective at storing water, nutrients, and microbiomes [69], all of which can improve the physical and chemical properties of soil and be beneficial to plant growth. Additionally, as small particulate materials are more readily degraded than those with large particles, fine particles of biochar can be more readily degraded by microbes than coarse ones, thereby affecting the nutrient cycle and other enzyme activities [69]. Nonetheless, intensive studies are still required to explore a long-term study of different biochar particles and BOF in saline–alkali soil in order to gain a more comprehensive understanding.

## 5. Conclusions

This study sought to determine the bacterial community and diversity of the root-associated soil of *M. crystallinum* and *A. cordifolia* in the saline–alkali land of China using high-throughput sequencing. The findings showed that adding biochar and BOF to soil positively influenced the growth of *M. crystallinum* and *A. cordifolia*, with B_BOF performing the best. Biochar and B_BOF also improved the physical and chemical properties and enhanced the nutrient concentration of the saline–alkali soil. Moreover, the diversity and community structure of the rhizosphere bacteria in the saline–alkali land was significantly affected by B_BOF, followed by BOF, and then biochar. The FB particle size was improved over the MB and CB. The current study provides guidance for the restoration of saline–alkali land in China. However, further studies are needed in order to focus on the long-term impacts of biochar and BOF on saline–alkali soil.

## Figures and Tables

**Table 1:** Effect of biochar and BOF on the physical and chemical properties of soil.

	CK	CB	MB	FB	BOF	B_BOF
EC	1.07 ± 0.08 a	0.93 ± 0.05 ab	0.92 ± 0.05 b	0.86 ± 0.05 b	0.85 ± 0.03 b	0.83 ± 0.04 b
pH	7.93 ± 0.07 a	7.86 ± 0.04 a	7.8 ± 0.08 a	7.82 ± 0.02 a	7.77 ± 0.1 a	7.78 ± 0.07 a
TP	0.71 ± 0.002 b	0.7 ± 0.006 bc	0.68 ± 0.011 c	0.74 ± 0.006 a	0.75 ± 0.008 a	0.76 ± 0.007 a
AP	7.18 ± 0.27 d	7.19 ± 0.27 d	7.31 ± 0.32 d	8.82 ± 0.32 c	12.4 ± 0.4 b	15.01 ± 0.56 a
TN	1.35 ± 0.06 c	1.47 ± 0.05 bc	1.46 ± 0.05 c	1.64 ± 0.06 b	0.97 ± 0.04 d	2.55 ± 0.11 a
AN	75.5 ± 2.8 cd	89.1 ± 3.66 ab	82.82 ± 2.38 bc	70.54 ± 2.17 d	68.86 ± 3.57 d	94.07 ± 4.85 a
OC	9.82 ± 0.49 c	13.69 ± 0.56 b	12.75 ± 0.72 b	13.57 ± 0.76 b	10 ± 0.49 c	22.57 ± 1.06 a
OM	17.12 ± 0.88 c	23.65 ± 1.28 b	22.17 ± 0.96 b	23.42 ± 1 b	17.22 ± 0.72 c	38.56 ± 1.99 a

Data are the means ± SD of three replicates. Values within a column followed by different lowercase letters are significantly different (*p* < 0.05).

## Data Availability

The data and results of this study are available upon reasonable request. Please contact the main author of this publication.

## Supplementary Materials

The following supporting information can be downloaded at: https://www.mdpi.com/article/10.3390/microorganisms10122310/s1, Figure S1: The pictures of *M. crystallinum* and *A. cordifolia*; Figure S2: Rarefaction curves depicting the number of OTUs with 97% similarity identified from different samples; Table S1: The relative ratio of fresh weight of aerial part; Table S2: Operational taxonomic unit richness and diversity indices of different samples; Table S3: top 38 genera attribution; Table S4: Correlation network analysis of microbial communities; Table S5: Node and degree in correlation network analysis.
