# Supplementary material for: Impact of Biochar and Bioorganic Fertilizer on Rhizosphere Bacteria in Saline–Alkali Soil
Source: Microorganisms. 2022 Nov 22;10(12):2310. doi: 10.3390/microorganisms10122310 (PMC9785793; doi:10.3390/microorganisms10122310)
Supplement: Supplementary file 1 [file microorganisms-10-02310-s001.zip › microorganisms-2028512-supplementary.pdf]

Supplementary Material

Table S1. The relative ratio of fresh weight of aerial part

	M. crystallinum (%)			*A. cordifolia* (%)		
	Coefficient compared to CK	Coefficient compared to FB	Coefficient compared to BOF	Coefficient compared to CK	Coefficient compared to FB	Coefficient compared to BOF
CK	100			100		
CB	127.70			125.45		
MB	132.43			121.82		
FB	164.86	100		179.09	100	
BOF	206.76	125.42	100	192.73	107.62	100
B-BOF	238.51	144.67	115.35	203.64	113.71	105.67

Figure S1. The pictures of *M. crystallinum* and *A. cordifolia*.

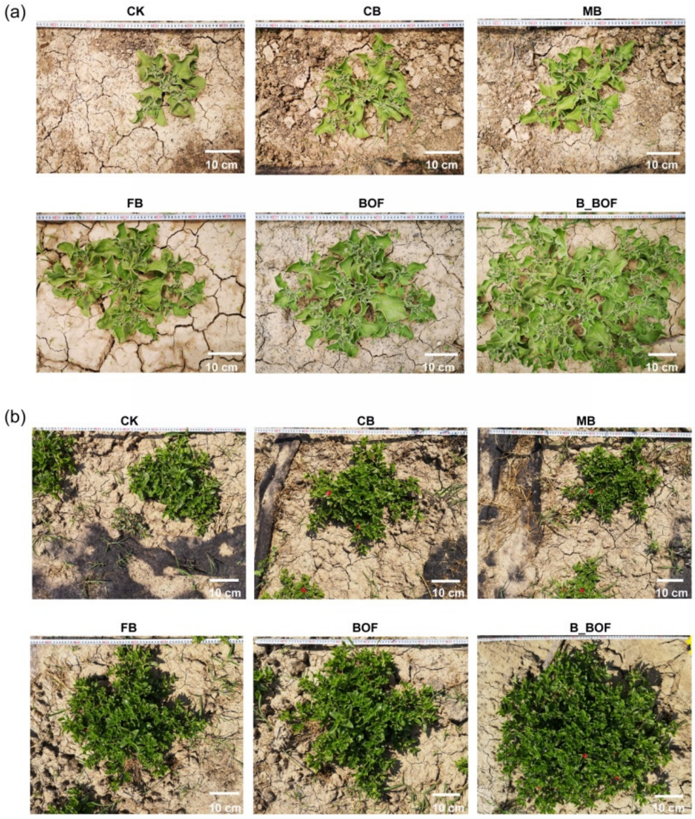

(a) *M. crystallinum*, (b) *A. cordifolia*.

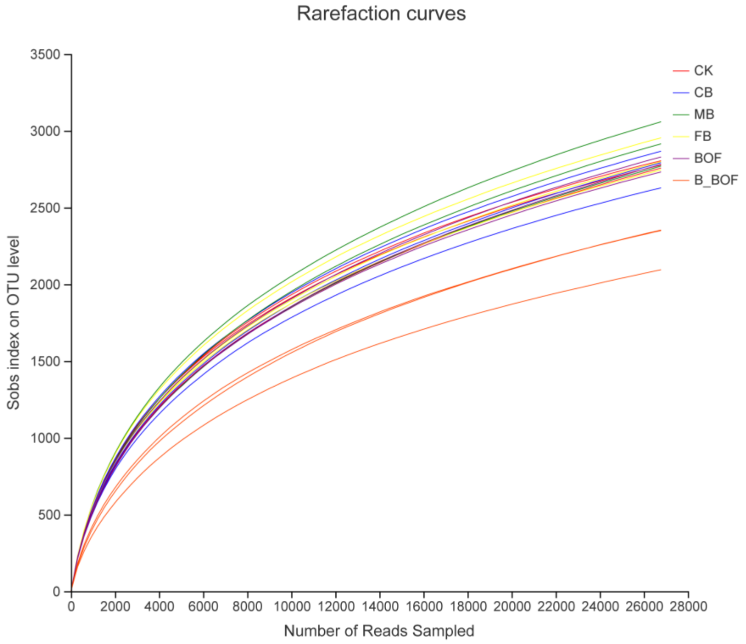

Figure S2. Rarefaction curves depicting the number of OTUs with 97% similarity identified from different samples

Table S2. Operational taxonomic unit richness and diversity indices of different samples

	sobs	shannon	simpson	ace	chao	coverage
CK	2780±9a	6.7±0.01a	0.0037±0.0001b	3868±141bc	3884±86ab	0.964±0.001ab
CB	2762±72a	6.65±0.07a	0.0041±0.0004b	4177±226abc	3978±171a	0.962±0.0014b
MB	2915±92a	6.72±0.09a	0.0037±0.0007b	4881±209a	4244±129a	0.959±0.0011b
FB	2833±54a	6.74±0.03a	0.0035±0.0002b	3987±52abc	4037±20a	0.962±0.0004b
BOF	2781±21a	6.61±0.07a	0.0049±0.0014b	4264±200ab	4071±83a	0.962±0.0008b
B-BOF	2266±65b	5.78±0.08b	0.0176±0.0021a	3287±110c	3369±95b	0.968±0.0011a

Table S3. top 38 genera attribution

Acidobacteria	norank_c__Subgroup_6
	RB41
	Bryobacter
Actinobacteria	Micromonospora
	Arthrobacter
	Blastococcus
	norank_c__Actinobacteria
	norank_f__67-14
	norank_o__Gaiellales

	Nocardioides
	Streptomyces
	norank_o__Microtrichales
	Marmoricola
	Actinomadura
	Nonomuraea
	Promicromonospora
Bacteroidetes	norank_f__Microscillaceae
Chloroflexi	norank_f__JG30-KF-CM45
	norank_c__KD4-96
	norank_f__A4b
	norank_o__SBR1031
	norank_c__TK10
	norank_f__Roseiflexaceae
	norank_f__Caldilineaceae
	norank_f__Anaerolineaceae
	OLB13
Firmicutes	Bacillus
	Paenisporosarcina
Gemmatimonadetes	norank_f__Gemmatimonadaceae
Patescibacteria	norank_f__Saccharimonadaceae
Proteobacteria	norank_f__Geminicoccaceae
	Skermanella
	unclassified_f__Burkholderiaceae
	MND1
	Pseudomonas
	Allorhizobium-Neorhizobium-Pararhizobium-Rhizobium
	Hydrogenophaga
Rokubacteria	norank_o__Rokubacteriales

Table S4. Correlation network analysis of microbial communities

	CK-B_BOF	CK-BOF	CK-FB
Node numbers	26	24	27
Edge numbers	104	45	42
Node average degree	8	3.75	3.11
Positive edges	67	26	23
Negative edges	37	19	19

The correlation network indices were calculated based on the top 30 genera. The average number of connections per node in the network, that is, the node connectivity.

Table S5. Node and degree in correlation network analysis

CK-BOF		CK-B_BOF		CK-FB	
Node_Name	Degree	Node_Name	Degr ee	Node_Name	Degr ee
Micromonospora	10	norank_f__JG30-KF-	14	norank_f__Gemmatimona	7

		CM45		daceae	
norank_c__Actinobacteria	7	norank_o__Gaiellales	13	norank_f__67-14	7
Blastococcus	7	norank_f__Geminicoccaceae	13	norank_f__Geminicoccaceae	6
norank_f__Gemmatimonadaceae	7	norank_f__Gemmatimonadaceae	13	norank_o__Gaiellales	5
norank_o__Rokubacteriales	6	norank_c__Actinobacteria	12	Candidatus_Entotheonella	5
norank_c__Gemmatimonadetes	5	Bryobacter	11	norank_o__Rokubacteriales	5
Microvirga	5	norank_o__Rokubacteriales	11	Bacillus	5
norank_f__Geminicoccaceae	5	norank_c__TK10	11	norank_f__Roseiflexaceae	5
RB41	4	Micromonospora	10	Gaiella	5
norank_f__Gemmataceae	4	norank_f__67-14	10	norank_o__Microtrichales	4
norank_c__TK10	4	norank_o__Microtrichales	9	Microvirga	4
norank_f__Anaerolineaceae	4	RB41	9	Arthrobacter	3
Nocardioides	3	norank_c__KD4-96	8	norank_c__Subgroup_6	2
norank_f__Caldilineaceae	3	Paenisporosarcina	7	norank_f__Caldilineaceae	2
norank_f__Roseiflexaceae	3	Bacillus	7	Blastococcus	2
norank_c__KD4-96	2	Skermanella	7	RB41	2
Bryobacter	2	norank_c__Subgroup_6	6	norank_f__AKYG1722	2
norank_o__SBR1031	2	norank_f__Microscillaceae	6	Skermanella	2
norank_f__67-14	2	norank_f__Roseiflexaceae	6	norank_f__JG30-KF-CM45	2
norank_c__Subgroup_6	1	Pseudomonas	5	Solirubrobacter	2
Skermanella	1	Nocardioides	4	norank_f__Gemmataceae	1
Marmoricola	1	Nonomuraea	4	norank_o__SBR1031	1
norank_f__A4b	1	Marmoricola	4	Streptomyces	1
norank_o__Gaiellales	1	unclassified_f__Burkholderiaceae	4	norank_c__KD4-96	1
		Streptomyces	3	norank_c__Actinobacteria	1
		norank_o__SBR1031	1	Marmoricola	1
				norank_c__TK10	1
